# Progress in the Relationship between Vitamin D Deficiency and the Incidence of Type 1 Diabetes Mellitus in Children

**DOI:** 10.1155/2022/5953562

**Published:** 2022-09-02

**Authors:** Lian-Ping He, Yu-Xin Song, Ting Zhu, Wei Gu, Chang-Wei Liu

**Affiliations:** ^1^School of Medicine, Taizhou University, Jiaojiang, 318000 Zhejiang, China; ^2^Children's Hospital of Nanjing Medical University, Nanjing, 210008 Jiangsu, China

## Abstract

Type 1 diabetes mellitus (T1DM) is an autoimmune disease, due to a large number of islet *β* cells damaged, resulting in an absolute lack of insulin, ultimately relying on insulin therapy. Vitamin D is a fat-soluble sterol derivative that not only participates in calcium and phosphorus metabolism but also acts as an immunomodulatory role by binding to nuclear vitamin D receptors to regulate the expression of transcription factors. Increasing evidence has shown that vitamin D has immunoregulation and anti-inflammatory effects, and it may play a role in T cell regulatory responses due to downregulation in the expression of cathepsin G and inhibition of CD4+ T cell activation and protection of *β* cells from immune attack and is beneficial in decreasing oxidative stress in T1DM patients. Epidemiologic evidence demonstrates involvement of vitamin D deficiency in T1DM pathogenesis, with the immune system improperly targeting and destroying its own islet *β* cells. In addition, polymorphisms in genes critical for vitamin D metabolism may increase the risk of islet autoimmunity and T1DM. In this paper, the relationship between vitamin D deficiency and the molecular mechanism of T1DM was discussed.

## 1. Introduction

Diabetes is one of the major chronic diseases and seriously threatens human health. The global diabetes prevalence in 20–79-year-olds in 2021 was estimated to be 536.6 million people (10.5%), rising to 783.2 million (12.2%) in 2045 [1]. Type 1 diabetes mellitus (T1DM) is a chronic autoimmune disease that tends to occur in children and adolescents, resulting from autoimmune degradation of pancreatic *β* cells leading to lifelong dependence on exogenous insulin. The number of children and adolescents (0–19 years old) with T1DM exceeded 1.1 million worldwide, and the number of new children and adolescents with T1DM was 128,900 per year, growing nearly 3% in the incidence rate [2]. The incidence of T1DM is increasing worldwide. Recent studies have shown that the overall population incidence of T1DM in China is 1.01 per 100,000 people per year [3], and the annual incidence of childhood with T1DM is about 2.02~5.3 per 100,000 [4]. The pathogenesis of T1DM has not been clearly defined so far, but it is generally thought to be due to multiple factors such as gene susceptibility and environment [5].

Serum vitamin D is bound to its binding protein, by which it is transported to the liver where it is converted to 25-hydroxy vitamin D [25(OH)D] by 25-hydroxylase; then, 25(OH)D is transformed to 1,25(OH)_2_D, the biological active form of vitamin D in the kidneys, through the action of the enzyme 25-hydroxyvitamin D-1alpha-hydroxylase (CYP27B1) [6]. The presence of CYP27B1 along with the vitamin D receptor (VDR) is found in several tissues [7, 8]. 1,25(OH)2D not only is involved in calcium and phosphorus metabolism but also acts in an immunomodulatory role by binding to nuclear VDRs to regulate the expression of transcription factors [9]. Vitamin D is closely related to the occurrence of autoimmune diseases [10, 11], which can play an important role in the pathogenesis of diabetes and glycemic control by inhibiting inflammatory and autoimmune responses, promoting insulin synthesis and secretion, and enhancing insulin sensitivity and vitamin D-related gene polymorphisms [12]. Vitamin D may play a role in T cell regulatory responses and defend *β* cells from immune attack [13]. Research progress on the relationship between vitamin D deficiency and the pathogenesis of T1DM is reviewed.

## 2. Pathogenic Relationship between Vitamin D Levels and T1DM

Vitamin D deficiency is closely related to the occurrence, development, and complications of T1DM [14–16]. T1DM patients were reported to have lower 25(OH)D levels compared to age-matched controls in children and adolescents [17–20]. A case-control study in northern India with Borkar et al. [21] suggested that 58% of T1DM had a 25(OH)D deficiency (25-OHD level < 20 ng/mL or <50 mmol/L), while the control group was only 32%. A similar previous study in our team found that serum 25(OH)D levels in patients with T1DM were 48.69 ± 15.26 nmol/L, compared with 57.93 ± 19.03 nmol/L in the control group [22]. According to the criteria of serum, 25(OH)D <50 nmol/L was insufficient and <30 nmol/L was deficient, and 49.66% of the patients had vitamin D insufficiency or deficiency, compared with only 30.51% in the control group. In the newly diagnosed T1DM children, the deficient and insufficient patients were 64.2%, while it was 41.60% in established T1DM. A systematic review analysis concluded that insufficiency or deficiency of 25(OH)D was associated with the development of T1DM in children [15]. Meanwhile, animal experiments have also shown that vitamin D deficiency in early life could accelerate T1DM in nonobese diabetic mice [23]. These studies suggest that deficiency of vitamin D will increase the risk of developing type 1 diabetes.

## 3. Pathogenesis of Autoimmune Response in T1DM

T1DM is a multifactorial chronic autoimmune disease in which blood glucose levels increase due to impaired secretion of islet *β* cells. It generally occurs in genetically susceptible individuals. During the development of T1DM, the immune system improperly targets and destroys its own islet *β* cells, resulting in progressive damage to insulin production and secretion function, and when T1DM is diagnosed, approximately 70–80% of *β* cell mass is destructed [24].

According to the genetic susceptibility to the disease and the targeting classification system [25–30], T1DM can be followed by four distinct stages:

- Stage 1 (islet autoimmunity). Subjects exhibit islet autoimmunity, as evidenced by the persistent presence of at least two islet autoantibodies [islet cell antibodies (ICAs), insulin autoantibodies (IAAs), glutamic acid decarboxylase 65 (GAD65), insulinoma-associated antigen 2 (IA-2), or zinc transporter 8 (ZnT8)]. During this stage, subjects remain normoglycemic and asymptomatic
- Stage 2 (abnormal glucose tolerance). Subjects maintain multiple islet autoantibody positivity and remain asymptomatic, but display dysglycemia, as evidenced by impaired fasting glucose, an abnormal oral glucose tolerance test (OGTT), or HbA1c ≥5.7%
- Stage 3 (symptomatic disease). Subjects experience the onset of clinical T1DM, which is often accompanied by symptoms such as polyuria, polydipsia, fatigue, weight loss, and diabetic ketoacidosis
- Stage 4. Established/long-term disease

## 4. Molecular Mechanism of Vitamin D Inhibiting T Cell Activation in T1DM

The process of pancreatic islet infiltration by immune cells represents the histological hallmark of the autoimmune destruction of *β* cells within the pancreatic islets. T1DM is mostly characterized by an inflammatory lesion [31], which is infiltrated by helper T cells (CD4+ or Th cells), cytotoxic T cells (CD8+), B lymphocytes, and macrophages, eventually leading to the destruction of islet *β* cells [32, 33], and CD8+ cytotoxic T lymphocytes are the most frequent among the islet infiltrating immune cells. Helper T cells also play an important role in T1DM pathophysiology [34]. Autoreactive CD8+ T cells recognize major histocompatibility complex (MHC) class I-restricted islet autoantigens on the *β* cell surface and exert cytotoxic effects through several effector mediators, particularly cytokines released by T helper type 1 (Th1) cells such as interferon (IFN)-*γ* [35]. T1DM patients exhibit defects in the ability of regulatory T cells (Treg cells) to suppress the activity and proliferation of autoreactive CD4+ and CD8+ T cells [36, 37].

Vitamin D has multiple roles in the body and can mediate immunomodulatory functions related to innate immune responses, antigen presentation, and adaptation to the immune system [38]. VDRs are expressed not only in islet *β* cells but also in immune cells, including activated T cells. Vitamin D has the effect of regulating islet *β* cells, increasing insulin sensitivity, and reducing the expression of inflammatory factor-induced apoptosis gene-related proteins to protect islet cells [39]. Vitamin D deficiency can cause abnormal glucose tolerance, impair the transcription of islet cell function genes [40], and increase the risk of developing type 1 diabetes and type 2 diabetes [41]. Vitamin D regulates the immune system by binding to receptors [42, 43].

Vitamin D can regulate T cells, promote CD4+ T cells to Th2 and Treg cell differentiation, reduce the production of Th1 and Th17 cells, and decrease the proportion of Th1/Th2. Vitamin D will also affect the production of cytokines, stimulating immune cells to release anti-inflammatory cytokines including IL-4, IL-10, and TGF-*β* while weakening the production of proinflammatory cytokines such as IFN-*γ*, IL-1*β*, IL-2, IL-6, IL-12, IL-17, IL-22, and TNF-*α* [44].

Autoantigens are presented by MHC at the beta-cell surface, and both CD4+ and CD8+ T cells isolated from peripheral blood could recognize epitopes in major islet autoantigens [32, 33]. Vitamin D promotes the maturation of monocytes into macrophages but at the same time decreases the ability of monocytes to present antigens to T cells by reducing the expression of superficial major histocompatibility complex MHC-II [45]. It also impairs dendritic cell maturation, leading to the formation of tolerant dendritic cells without surface MHC molecules and consequently being unable to present antigens [46]. Impaired antigen presentation by antigen-presenting cells (APCs) leads to unresponsive T cells, thereby inhibiting and/or impinging B cell proliferation, plasma cell differentiation, formation of memory *β* cells, and production of autoantibodies [47].

**Figure 1:**
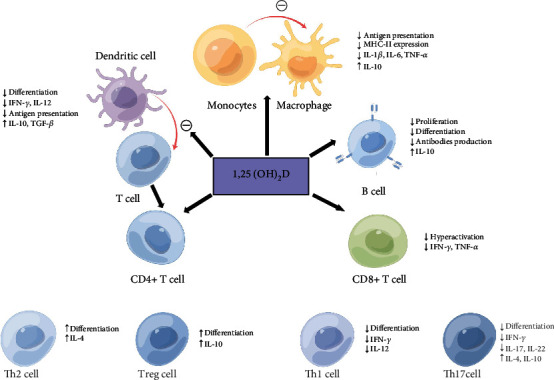
A review of anti-inflammatory and immunoregulation of 1,25(OH)2D on immune systems [27, 44]. ↓ or ㊀ represents downregulation, and ↑ represents upregulation. IFN-*γ*, interferon gamma; IL-1*β*, interleukin 1*β*; IL-2, interleukin 2; IL-4, interleukin 4; IL-6, interleukin 6; IL-10, interleukin 10; IL-12, interleukin 12; IL-17, interleukin 17; IL-22, interleukin 22; MHC-II, major histocompatibility complex-II; TGF-*β*, transforming growth factor-beta; TNF-*α*, tumor necrosis factor-alpha.

When vitamin D is deficient, the inhibitory effect on T cells is weakened and the chemokines secreted by T cells increase, which can recruit immune cells to participate in autoimmune attacks on *β* cells [48]. At the same time, cytokines released by immune cells can upregulate the expression of MHC-I and MHC-II and adhesion molecules on islet *β* cells, promoting the interaction of *β* cells with cytotoxic T cells and inducing apoptosis. The continued progression of vitamin D deficiency is also accompanied by impaired proliferative activity of T cells and imbalances in the proportion of regulatory T cells (CD4+) and CD8+ T cells while vitamin D supplementation normalizes the proliferative activity of T cells and the proportion of T cell subsets.  Figure 1 displays the immunomodulatory and anti-inflammatory actions of 1,25(OH)_2_D.

The immunomodulatory effects of vitamin D, promoting immune tolerance and induction of T cell anergy, impinging on B cell activity and antibody production, and reducing the inflammatory response, suggest the therapeutic potential of calcitriol in autoimmune diseases including T1DM.

## 5. Mechanism of Vitamin D Downregulating CatG Expression in T1DM

Numerous studies in vivo and in vitro have revealed potent anti-inflammatory actions of vitamin D that affect the major cellular players associated with autoimmune disease [49–51]. T1DM is an autoimmune disease attributed to progressive injury of islet *β* cells mediated by T cells. Cathepsin G (CatG) is involved in the antigen presentation of proinsulin. It was demonstrated by Zou et al. that CatG could impact the activation of CD4+ T cells in nonobese diabetic (NOD) mice. During the pathogenesis of diabetes, the expression level of CatG in NOD mice gradually increased and the CD4+ T cells were gradually activated, resulting in more Th1 cells and fewer Th2 and Treg cells. Treatment with a CatG-specific inhibitor could reduce the blood glucose level, improve the function of islet *β* cells, and reduce the activation of CD4+ T cells [52]. A new study further discovered that vitamin D supplementation could improve pancreatic *β* cell function and suppress immunological and inflammatory reactions in the T1DM mice. The overexpression of CatG in diabetes tissue samples was documented and then showed that vitamin D supplementation normalizes the islet immune microenvironment through downregulating CatG expression in T1DM mice. Experiments in vitro subsequently elucidated that vitamin D supplementation could impede CD4+ T activation by downregulating CatG expression and thereby enhancing pancreatic *β* cell function [53]. Therefore, vitamin D could downregulate the expression of CatG to inhibit CD4+ T cell activation and prevent the destruction of islet *β* cells by immune cells and is expected to play a therapeutic role as an immunomodulator in type 1 diabetes.

## 6. Vitamin D Inhibiting Oxidative Stress in T1DM

Environmental factors can also trigger the onset of T1DM and influence its progression. The factors include viral infections, toxins, reactive oxygen species (ROS), and chronic inflammation, which are triggers of endoplasmic reticulum (ER) stress. ER stress initiates the unfolded protein response (UPR), which acts through inositol-requiring protein-1 (IRE1), protein kinase RNA-like endoplasmic reticulum kinase (PERK), and activating transcription factor-6 (ATF6), all of which are localized to the ER membrane and respond to stress by transmitting ER signals to the cytoplasm and nucleus. Although UPR initially attempts to mitigate ER stress, if the stress is prolonged or severe, it switches from a proapoptotic to a problematic response [54, 55]. It has been reported that modulating the unfolded protein response (UPR) in *β* cells of nonobese diabetic (NOD) mice by deleting the UPR sensor IRE1*α* prior to insulitis induced transient dedifferentiation of *β* cells, resulting in substantially reduced islet immune cell infiltration and *β* cell apoptosis, and IRE1*α*-deficient mice exhibited significantly fewer cytotoxic CD8+ T cells in their pancreata, and adoptive transfer of their total T cells did not induce diabetes [54]. Inflammation in pancreatic islets occurs early during the pathogenesis of T1DM. Proinflammatory signaling in *β* cells might be conducive to the immunogenicity of *β* cells. The recent study supports the contention that inflammatory signaling in *β* cells promotes autoimmunity during T1DM progression [55]. It has been shown that *β* cells of prediabetic NOD mice display dysfunction and overt ER stress that may be driven by NF-*κ*B signaling, and strategies that attenuate pathways leading to ER stress may preserve *β* cell function in T1DM [56].

Vitamin D may reduce oxidative markers such as superoxide dismutase (SOD) in T1DM, which have high insulin and C-peptide levels in the treatment group compared to other groups [57]. Interestingly, in a study, calcitriol plays a prominent role in suppressing ROS, regenerating glutathione (GSH), and reversing the pro-atherogenic phenotype in human umbilical vein endothelial cells caused by hyperketonemia [58]. One main source of oxidative stress in pancreatic *β* cells appears to be the reactive oxygen species producer NADPH oxidase (NOX) enzyme, which has a role in *β* cell death [59].

Presumably, vitamin D supplementation may be beneficial in reducing oxidative stress in patients with T1DM. Further studies need to be carried out to demonstrate the effect of vitamin D in the prevention of T1DM where inducing *β* cell dedifferentiation, prior to insulitis, allows these cells to escape immune-mediated destruction.

## 7. Polymorphisms of Vitamin D Metabolism Genes and T1DM

Different polymorphisms of genes, such as encoding vitamin D hydroxylases and VDR, may influence the risk of islet autoimmunity and T1DM [27]. Serum vitamin D levels are influenced by variants in genes involved in the synthesis, transport, hydroxylation, and degradation of vitamin D. A relationship between single-nucleotide polymorphisms (SNPs) of the gene encoding the vitamin D 25-hydroxylase in CYP2R1 and T1DM was studied. A case-control study of 252 children less than 20 years old found that vitamin D deficiency was more prevalent in children with T1DM than in healthy controls. CYP2R1 rs12794714 and rs10766196 polymorphisms were associated with a high risk of T1DM, and polymorphisms in vitamin D metabolism could lead to susceptibility to T1DM in Korean children [60]. Another similar study showed a cumulative effect of SNPs at the CYP2R1 (rs2060793), DHCR7 (rs12785878), GC (rs2282679), and CYP24A1 (rs6013897) loci on the susceptibility to type 1 diabetes [61]. Tangjittipokin et al. [62] found SNPs and T1DM in CYP2R1 (rs10741657) (GA, OR: 1.83, 95% CI: 1.01–3.31; *p* = 0.04). CYP27B1 (rs4646536) was negatively associated with 25(OH)D) levels, and CYP27B1 (rs4646536) and GC (rs2282679) were positively associated with TNF-*α* levels. Hussein et al. [63] reported that the GG genotype of CYP2R1 (SNP rs10741657) or CC genotype of CYP27B1 (SNP rs10877012) increased the risk of developing T1D in Egyptian children.

A potential role of single-nucleotide polymorphisms (SNPs) of the VDR gene in T1D, including FokI (rs10735810), ApaI (rs7975232), TaqI (rs731236), and BsmI (rs1544410), has been reported. Results by Rasoul et al. [64] demonstrated a significant effect of two VDR gene polymorphisms (FokI and TaqI) (FokI, C>T, rs10735810, and TaqI, C>T, rs731236) on the genetic susceptibility of T1DM in Kuwaiti Arabs; meanwhile, the VDR gene ApaI (G>T, rs7975232) and BsmI (A>G, rs1544410) polymorphisms were not associated with T1DM.

Tangjittipokin et al. [62] found that VDR gene-related variations of ApaI (rs7975232), TaqI (rs731236), and BsmI (rs1544410) were negatively associated with vitamin D and IL-10 levels in children with T1DM. Norris et al. [65] showed that higher serum 25(OH)D levels are associated with a lower risk of islet autoimmunity in children at increased genetic risk of T1DM, and the association between childhood 25(OH)D status and islet autoimmunity was modified by the ApaI (rs7975232) SNP in VDR (interaction *p* = 0.0072), where for each additional minor allele, higher 25(OH)D concentrations were associated with a greater reduction in islet autoimmunity risk. Therefore, vitamin D and VDR may play a combined role in the development of islet autoimmunity among children with increased genetic risk for T1DM.

The relationship between inherited variation in vitamin D genes and diabetes has been addressed in recent reviews and meta-analyses [66–68]. However, there were many studies that did not confirm these results [69]. There was a lack of association of vitamin D receptor gene polymorphisms of VDR genes including FokI, (rs10735810), ApaI (rs7975232), TaqI (rs731236), and BsmI (rs1544410) with susceptibility to T1DM in the Portuguese population [70]. Thorsen et al. [71] did not find an association between SNPs in CYP2R1, CYP27B1, VDR, and GC and the risk of T1DM in a juvenile Danish population, though 25(OH)D levels were associated with variants in the GC gene. A systematic review and meta-analysis of genetic evidence showed no large effect of a genetically determined reduction in serum 25(OH)D concentrations by selected polymorphisms on T1DM risk, despite the strong association seen in some observational studies [72].

These studies suggest that SNPs in genes critical for the synthesis, transport, and action of vitamin D may affect the risk of T1D development. The polymorphisms may be associated with decreased VDR, 25-hydroxylase, and 1ɑ-hydroxylase activity and expression. In order to investigate the relationship between T1DM pathogenesis and SNPs in genes involved in vitamin D metabolism, more prospective studies are needed.

## 8. Effects of Vitamin D Interventions on T1DM

Vitamin D deficiency can affect T1DM, and low vitamin D levels are strongly associated with ketoacidosis in children with new-onset type 1 diabetes [73]. Even in patients with T1DM who have been diagnosed for several months, vitamin D deficiencies are still a cause for concern [22, 74]. Studies have shown that by simply regulating the intake of vitamin D in children with T1DM without changing the amount of insulin, glycated hemoglobin (HbA1c) can also be better controlled [75]. From another perspective, vitamin D deficiency can affect blood glucose control in children with T1DM. Studies have revealed that T1DM children with vitamin D deficiency are more likely to have hypoglycemia and metabolic diseases [76]. The possible role of vitamin D supplementation, as an additional therapy, to increase glycemic control and insulin sensitivity opens new perspectives to increase the control of the disease and improve the health of these patients [77]. In a double-blind randomized controlled study by Treiber et al. [78], vitamin D supplementation in T1DM children was shown to enhance the inhibitory capacity of Treg cells and increase the proportion of Treg cells, while reducing the need for fasting blood glucose, HbA1c, and exogenous insulin. This suggests that vitamin D supplementation can affect the onset and development of T1DM and that patients with T1DM with inadequate vitamin D levels should be treated with vitamin D.

Regular vitamin D supplementation in early infancy may reduce the risk of T1DM [79–81]. A birth cohort study of more than 10,000 children found similar protective effects [82]. Therefore, in the first years of life, vitamin D deficiency should be promptly diagnosed and appropriately treated, especially in children at high genetic risk for T1DM, which is defined by a family history of T1DM and islet autoantibodies and/or human leukocyte antigen (HLA) positivity. Vitamin D should be considered an additional therapy.

## 9. Conclusion

T1DM is an autoimmune disease characterized by the T cell-mediated destruction of insulin-producing *β* cells in pancreatic islets. Vitamin D has immunomodulatory and anti-inflammatory actions, and its deficiency may play a role in the pathogenesis of T1DM, indicating hypovitaminosis D as an important environmental factor for the development of the disease. Vitamin D has a role in T cell regulatory response by protecting *β* cells from immune attack and is beneficial in reducing oxidative stress in patients with T1D. However, studies on vitamin D supplementation and preservation of *β* cell function in T1DM have no conclusion. In the future, large-scale prospective trials are needed to fully evaluate the role of vitamin D as a disease modifier for T1DM.

## Data Availability

The datasets generated and analyzed during the current study are available from the corresponding author on reasonable request.
